# Nanostructured branched Y-DNA promotes antitumor immunity through dual activation of cGAS/STING and TLR9

**DOI:** 10.1007/s12272-026-01607-y

**Published:** 2026-03-18

**Authors:** Jin Kyung Seok, Jung In Jee, Jong Han Oh, So Yeon Ahn, Hana Cho, Young Eun Yang, Minhyuk Kim, Hyung-Joo Kwon, Yong-Yeon Cho, Hye Suk Lee, Soong Ho Um, Han Chang Kang, Joo Young Lee

**Affiliations:** 1 College of Pharmacy, The Catholic University of Korea, Bucheon, 14662 Korea; 2 School of Chemical Engineering, Sungkyunkwan University, Suwon, 16419 Korea; 3 Department of Biopharmaceutical Science, Soonchunhyang University, Asan, 31538 Korea; 4 Department of Microbiology, College of Medicine, Hallym University, Chuncheon, 24252 Korea; 5 Present Address: Department of Chemical Engineering, Columbia University, New York, 10027 USA

**Keywords:** Combination therapy, Nanomedicine, Toll-like receptor, cGAS, Immune activation, Tumor microenvironment

## Abstract

Effective activation of innate and adaptive immunity remains a major challenge in cancer immunotherapy. In this study, we aimed to develop an immunostimulatory oligonucleotides for antitumor immunotherapy. We designed branched Y-shaped DNA (Yb-DNA) nanoparticles complexed with a reducible polycationic carrier (RPC-bPEI_0.8kDa_), which we named YbNano, to enhance intracellular delivery and immune activation. The immunostimulatory activity of YbNano was determined in mouse bone marrow-derived dendritic cells. The efficacy of YbNano for antitumor immunotherapy was investigated using a melanoma allograft mouse model and a lung metastasis of breast cancer mouse model. YbNano exhibited uniform particle morphology and efficient cellular uptake in dendritic cells. YbNano induced robust expression of type I interferon (IFN-β), interleukin-12 (IL-12), chemokines such as CXCL10, and costimulatory molecules (CD80, CD86) in dendritic cells, promoting CD8⁺ T cell differentiation. In preclinical cancer models, intravenous administration of YbNano potentiated the efficacy of anti-PD-L1 therapy in B16F10 melanoma-bearing C57BL/6 mice and enhanced the therapeutic outcomes of doxorubicin or anti-PD-L1 treatment in suppressing lung metastasis in 4T1 breast cancer-bearing BALB/c mice. YbNano functions as a dual activator of cGAS/STING and TLR9, orchestrating dendritic cell activation and amplifying downstream innate and adaptive immune responses in tumor microenvironment. Collectively, these findings suggest that YbNano represents a rationally engineered, multifunctional nucleic acid-based immunotherapeutic agent with the potential to modulate the tumor microenvironment and to augment responses when used in combination cancer therapy.

**Supplementary Information:**

The online version contains supplementary material available at 10.1007/s12272-026-01607-y.

## Introduction

Immune evasion is a critical hallmark of cancer progression, enabling tumors to escape immune surveillance through multiple molecular and cellular mechanisms including the increased resistance to immune effector molecules such as granzyme B and the acquisition of cancer stemness (Cullen et al. 2010; Chiang et al. 2024; Galassi et al. 2024). Therefore, immune evasion blockade strategies, including immune checkpoint inhibitors, 20S proteasome inhibitors, and regulation of long intergenic non-coding RNAs, have been suggested as promising immunotherapies for cancer (Lee et al. 2016; Atta et al. 2023; Eldash et al. 2023). Recent evidence suggests that by harnessing the immune system, long-lasting diseases can be controlled and treated (Seok et al. 2021). However, many patients do not exhibit T cell infiltration, have a cold tumor microenvironment characterized by a high density of immunosuppressive cells, and do not respond to the U.S. Food and Drug Administration (FDA)-approved immune checkpoint blockade (ICB) antibodies (Sharma et al. 2017). Therefore, strategies that create an improved response to checkpoint blockade and a hot tumor microenvironment that prevents tumor re-challenge are required for cancer immunotherapy (Wu et al. 2024).

Innate immune sensing, mediated by pattern-recognition receptors (PRRs) such as Toll-like receptors (TLRs) and cytosolic DNA sensors including the cGAS/STING pathway, is central to initiating effective antitumor immunity (Koo et al. 2015; Seok et al. 2023). Activation of these pathways in dendritic cells (DCs) induces the expression of pro-inflammatory cytokines, chemokines, and costimulatory molecules, promoting the differentiation of naïve T cells into effector subsets, and further amplifying the cytolytic activity of cytotoxic T lymphocytes and natural killer (NK) cells, while IFN-γ production enhances both innate and adaptive immune functions (Gupta et al. 2022). Coordinated engagement of multiple immune pathways therefore represents a promising strategy to overcome the limitations of single-receptor targeting immunotherapies, particularly in tumors with low immunogenicity (Hu et al. 2024).

Nucleic acid-based immunostimulatory agents have emerged as versatile modulators of innate immunity (Klinman 2004). Our previous research reported that a ligated form of X-shaped DNA alleviated the symptoms of atopic dermatitis and improved the therapeutic efficacy of doxorubicin in colon cancer treatment (Koo et al. 2015; Yang et al. 2018, 2019). DNA oligonucleotides are highly anionic and soluble, which limits their cellular uptake and promotes rapid extracellular clearance (Vrignaud et al. 2011). DNA transfection reagents, such as cationic liposomes (Nabel et al. 1993) or cationic polymers (Boussif et al. 1995; Tang et al. 1996) have been used to form the complex with anionic DNAs to increase the cellular delivery of DNA (Ruponen et al. 1999).

In this study, we aimed to develop a nucleic acid-based immunostimulatory agent, which could be applicable for antitumor immunotherapy. We designed nanoparticles of immunostimulatory branched Y-shaped DNA (Yb-DNA) complexed with reducible polycation (RPC)-bPEI, which exhibits low cytotoxicity and efficient material delivery. By improving intracellular delivery of DNA and coordinated activation of TLR9 and cGAS/STING pathways, this platform provides a basis for enhancing antitumor immune responses and modulating the tumor microenvironment, supporting its potential use in combination cancer immunotherapy.

## Materials and methods

### Animals

Male C57BL/6 mice and female BALB/c mice were obtained from DBL (Eumseong, Korea) and acclimated under specific pathogen-free conditions in an animal facility for at least a week before the experiments. A temperature (23 ± 3 °C) and relative humidity (40–60%)-controlled room was used to house the mice. All the animals were received humane care based on the “Guide for the Care and Use of Laboratory Animals” criteria outlined by the National Academy of Sciences in the National Institutes of Health publication. The animal experiments and study design were approved by the Institutional Animal Care and Use Committee (IACUC) of the Catholic University of Korea (permission number: 2019-027, 2020-004).

### Cell culture

The preparation of primary dendritic cells (DCs) was conducted as described previously (Yeon et al. 2017). Bone marrow cells were isolated from the mice and cultured in RPMI1640 medium (Gibco, Waltham, MA) containing 10%(v/v) heat-inactivated fetal bovine serum (FBS; Life Technologies; Grand Island, NY), 50 μM 2-mercaptoethanol (Gibco), 100 units/ml penicillin (Gibco), 100 μg/ml streptomycin (Gibco), 2 mM glutamine (Gibco), and 3% J558L hybridoma cell culture supernatant containing granulocyte–macrophage colony-stimulating factor (GM-CSF) for 6 days. Non-adherent cells were used as bone marrow-derived dendritic cells (BMDCs). Human embryonic kidney cells (HEK293T) were cultured in Dulbecco’s modified eagle medium (DMEM) supplemented with 10% FBS, 100 units/ml penicillin, and 100 μg/ml streptomycin. Mouse skin melanoma cells (B16/F10) were also cultured in the same manner. Mouse breast cancer cells (4T1) were cultured in RPMI-1640 medium supplemented with 10% FBS, 100 units/ml penicillin, and 100 μg/ml streptomycin. TLR9 knockout (KO) bone marrow cells were obtained from Balb/c TLR9 KO mice as previously described (Kim et al. 2018b). THP-1 Lucia ISG (THP-1 wild-type) and THP-1 cGAS KO cells (InvivoGen, San Diego, CA) were maintained with normocin (100 µg/ml, InvivoGen) and zeocin (100 µg/ml, InvivoGen), with the THP-1 cGAS KO cells additionally supplemented with blasticidin (10 µg/ml, InvivoGen). The cells were maintained at 37 °C in a 5% CO_2_/air environment.

### Reagents

Dynasore, cytochalasin D, and chloroquine were purchased from Sigma Aldrich (St. Louis, MO). Bafilomycin A1 was obtained from Calbiochem (Billerica, MA). ODN2395 that was used as a TLR9 agonist was obtained from InvivoGen. G3-YSD was obtained from InvivoGen. LPS was acquired from Biological Laboratory Inc. (Campbell, CA).

### Fabrication of branched Y-shaped DNA

The formation of branched Y-shaped DNA oligonucleotides was performed similarly as described previously (Um et al. 2006; Yang et al. 2018, 2019). Branched Y-shaped DNA (Yb-DNA) was fabricated by mixing the same molar amount of Yb1, Yb2, Yb3, Yb4, Yb5, and Yb6 oligonucleotide strands each with an aqueous 200 mM sodium chloride solution. The sequence information of the oligonucleotides is shown in Supplementary Fig. 1. The mixtures were incubated at 95 °C for 5 min and annealed from 95 to 4 °C with a temperature ramp of − 0.5 °C/30 s. The oligonucleotides utilized in this study were procured from Integrated DNA Technologies (Coralville, IA).

### Preparation of Yb-DNA/RPC nanoparticles (YbNano)

The synthesis of RPC-bPEI_0.8kDa_ was performed as described previously (Kang et al. 2011). RPC-bPEI_0.8kDa_ (2 μg/10 μL) was combined with Yb-DNA (1 μg DNA/10 μL) in 4-(2-hydroxyethyl)-1-piperazineethanesulfonic acid (HEPES) buffer (pH 7.4, 20 mM, Gibco) for 30 min at room temperature. The resultant complexes were used in further experiments. The weight ratio (WR; reducible polycation weight per DNA weight) was varied (i.e., WR1, WR2, and WR4). Condensation of Yb-DNA with RPC-bPEI_0.8kDa_ was monitored using a gel electrophoresis assay, using a 0.8%(w/v) agarose gel supplemented with ethidium bromide. The gel was run at 100 V constantly in tris–acetate-ethylenediaminetetraacetic acid (TAE) buffer for 10 min. Uncomplexed Yb-DNA or exposed Yb-DNA in the polyplexes was detected using a UV illuminator. The surface charges and particle sizes of the Yb-DNA/RPC-bPEI_0.8kDa_ complexes were measured at room temperature in the HEPES solution (20 mM, pH 7.4) using a zeta potential and size analyzer (ELS-Z; Photal Otsuka Electronic Co., Osaka, Japan) at 677 nm and a constant 90° angle. The morphology and size of YbNano were examined using transmission electron microscopy (JEM-1010, JEOL, Tokyo, Japan). TEM images were acquired at a scale bar of 100 nm.

### Enzyme-linked immunosorbent assay (ELISA)

ELISA was performed as described previously (Yang et al. 2023). Concentrations of interferon (IFN)-β, IL-12 p40, IFN-γ, and C-X-C motif chemokine ligand (CXCL)-10 (R&D Systems, Minneapolis, MN) in the culture supernatants and 2′3′-cyclic guanosine monophosphate-adenosine monophosphate (2′3′-cGAMP) (Cayman, Ann Arbor, MI) in the cells were determined using ELISA according to the manufacturer’s instructions. Plates were read at 450 nm wavelength with a microplate reader (Molecular Devices, San Francisco, CA). The concentration ranges of the standard curves were 15.6 to 1,000 pg/ml for IFN-β, 62.5 to 4,000 pg/ml for IL-12 p40, 31.2 to 2,000 pg/ml for IFN-γ, 7.8 to 500 pg/ml for CXCL-10, and 6.1 pg/ml to 100 ng/ml for 2′3′-cGAMP. Samples were accurately diluted to ensure measurement within the standard curve ranges.

### T cell activation by YbNano-treated dendritic cells

T cells were isolated from the spleens of C57BL/6 mice using anti-mouse CD8a microbeads and MACS LS columns according to the manufacturer’s instructions (Miltenyi Biotec, Auburn, WA). After bone marrow-derived DCs (BMDCs) were stimulated with ovalbumin (10 μg/ml) in the presence or absence of YbNano for 18 h, the cells were co-cultured with CD8a-positive T cells for 3 days (DC:T cells ratio, 1:10). The concentrations of cytokines in the culture supernatants were determined using ELISA.

### Reverse transcription and quantitative polymerase chain reaction (qRT-PCR) analysis

qRT-PCR analysis was performed as described previously (Joung et al. 2011). Total RNAs were isolated using trizol reagent (Invitrogen, Carlsbad, CA). RNAs were reverse transcribed using ImProm-II™ Reverse Transcriptase (Promega, Madison, WI) and amplified with IQ™ SYBR® Green Supermix (Bio-Rad, Hercules, CA) using an IQ™5 (Bio-Rad) for quantitative real-time PCR. The primers were: IFN-β, 5′-TCCAAGAAAGGACGAACATTCG-3′ (forward) and, 5′-TGAGGACATCTCCCACGTCAA-3′ (reverse); IL-12, 5′-GAAGTTCAACATCAAGAGCAGTAG-3′ (forward) and 5′-AGGGAGAAGTAGGAATGGGG-3′ (reverse); CXCL10, 5′-AGAACGGTGCGCTGCAC 3′ (forward) and, 5′ CCTATGGCCCTGGGTCTCA-3′ (reverse); CD86, 5′-TGTTTCCGTGGAGACGCAAG-3′ (forward) and 5′- TTGAGCCTTTGTAAATGGGCA-3′ (reverse); CD80, 5′-ACCCCCAACATAACTGAGTCT-3′ (forward) and 5′-TTCCAACCAAGAGAAGCGAGG-3′ (reverse); CD40, 5′- GCTATGGGGCTGCTTGTTGA-3′ (forward) and 5′- ATGGGTGGCATTGGGTCTTC-3′ (reverse); β-actin, 5′-TCATGAAGTGTGACGTTGACATCCGT-3′ (forward) and 5′-TTGCGGTGCACGATGGAGGGGCCGGA-3′ (reverse). The specificity of the amplified PCR products was assessed using a melting curve analysis. Gene-expression fold induction was calculated after each target gene’s mRNA level was normalized to the β-actin level in the corresponding sample.

### Flow cytometric analysis

CD80, CD86, and CD40 surface molecules were stained with FITC-conjugated anti-mouse antibodies against each protein (BD Pharmingen, San Jose, CA) for 3 h at 4 °C. Fluorescent Yb-DNA was prepared from the Yb1 oligonucleotide bearing a 5′-carboxyfluorescein (FAM) modification. BMDCs were treated with fluorescent Yb-DNA or YbNano containing fluorescent Yb-DNA for 4 h. After washing the cells with staining buffer (BD Pharmingen), the cells were analyzed using a FACSCanto flow cytometer (BD Biosciences, San Jose, CA).

### Confocal microscopy analysis

Confocal microscopy was performed using ZEN 2011 imaging system (Carl Zeiss, Oberkochen, Germany) as described previously (Jang et al. 2023). BMDCs were treated with fluorescent Yb-DNA or YbNano containing fluorescent Yb-DNA for 4 h. For inhibition studies, BMDCs were pretreated with several endocytosis inhibitors for 30 min before treatment with Yb-DNA or YbNano. Intracellular localization was then examined using confocal microscopy.

### Transfection of plasmid DNA or siRNA

Transfection of plasmid DNA or siRNA was conducted as described previously (Seok et al. 2025). SuperFect reagent (Qiagen, Hilden, Germany) was used for plasmid DNA transfection according to the manufacturers’ instruction. NF-κB-dependent luciferase reporter plasmid was provided by Dr. Frank Mercurio (Signal Pharmaceuticals, San Diego, CA). IFN-β PRD III-I promoter-luciferase reporter plasmid was a kind gift from Katherine Fitzgerald (University of Massachusetts Chan Medical School, Worcester, MA). pCMV-Myc-STING plasmid was a kind gift from Dr. Andrew Bowie (Trinity College Dublin, Dublin 2, Ireland). siRNAs were purchased from Dharmacon (A-055608-13-0020, A-055608-15-0020, Lafayette, CO) and transfected into the cells with Accell siRNA delivery medium (Dharmacon) according to standard methods.

### Co-precipitation assay for Yb-DNA binding to TLR9 or cGAS

This study was performed as described previously (Akira et al. 2006). HEK293T cells were transfected with the pcDNA3.1-mTLR9-Myc/His plasmid or pUno1-cGAS-3xHA plasmid (InvivoGen). Cell lysates were incubated with biotinylated Yb-DNA for 2 h and precipitated with NeutrAvidin (NA) beads (Thermo Scientific, Rockford, IL) for 4 h at 4 °C on a rocker. The precipitated complexes were washed three times and solubilized using Laemmli sample buffer. Sodium dodecyl sulfate–polyacrylamide gel electrophoresis (SDS-PAGE) was used to resolve the solubilized proteins and the proteins were analyzed by immunoblotting as described previously (Jeong et al. 2014). Anti-Myc antibodies were purchased from Cell Signaling Technology (Beverly, MA, USA). Anti-HA antibodies from Roche (Rotkreuz, Switzerland).

### A mouse melanoma allograft model

B16F10 cells were trypsinized, resuspended in DMEM, and injected into the left flank of 8-week-old C57BL/6 mice (1 × 10^6^ cells/mouse). When tumor volumes were reached to approximately 10 mm^3^, mice were randomly divided into treatment groups. YbNano (10 μg Yb-DNA/100 μL phosphate-buffered saline) was intravenously administered every 3 days, starting at day 8. An equivalent amount of PBS was delivered as a vehicle control. Tumor size was measured every 3 days using a digital caliper and tumor volume was calculated using the following equation: (length × width^2^) × 0.5. The mice were euthanized on day 17, and tumors were harvested, weighed, and processed for further experiments. To check for toxicity, mouse behavior and body weight changes were monitored. No death was observed during the experiment period.

### A breast cancer lung metastasis mouse model

4T1 cells (5 × 10^5^ cells/100 μL) were injected into the tail veins of the BALB/c mice. The mice were intravenously administered with YbNano, RPC, or HEPES solutions through the tail vein 7 days after tumor inoculation. On the same day, doxorubicin, anti-PD-L1 antibody, or PBS was administered. Body weights were assessed every 3 days. After all the animals were euthanized, the lungs were harvested, their weights were recorded, and the number of lung metastases were counted.

### Histological analysis

The histological analysis was performed as described previously (Yang et al. 2021). Tumor sections were stained with hematoxylin and eosin and with antibody for F4/80 (Cell Signaling Technology) or NKp46 (Biorbyt, Cambridge, UK). A terminal deoxynucleotidyl transferase dUTP nick-end labeling (TUNEL) assay was performed on tissue sections using a chromogenic kit (Merck, Kenilworth, NJ).

### Statistical analysis

Statistical analysis was performed using the GraphPad Prism7 software (GraphPad Software, San Diego, CA). All the data were expressed as mean ± standard error of the mean (SEM) and analyzed for normality using the Kolmogorov–Smirnov test before applying parametric statistical tests. One-way analysis of variance (ANOVA) followed by Tukey’s multiple comparison test were then conducted. P-values < 0.05 were considered statistically significant.

## Results

### Development of immune-stimulatory branched Yb-DNA nanoparticle (YbNano)

**Fig. 1:**
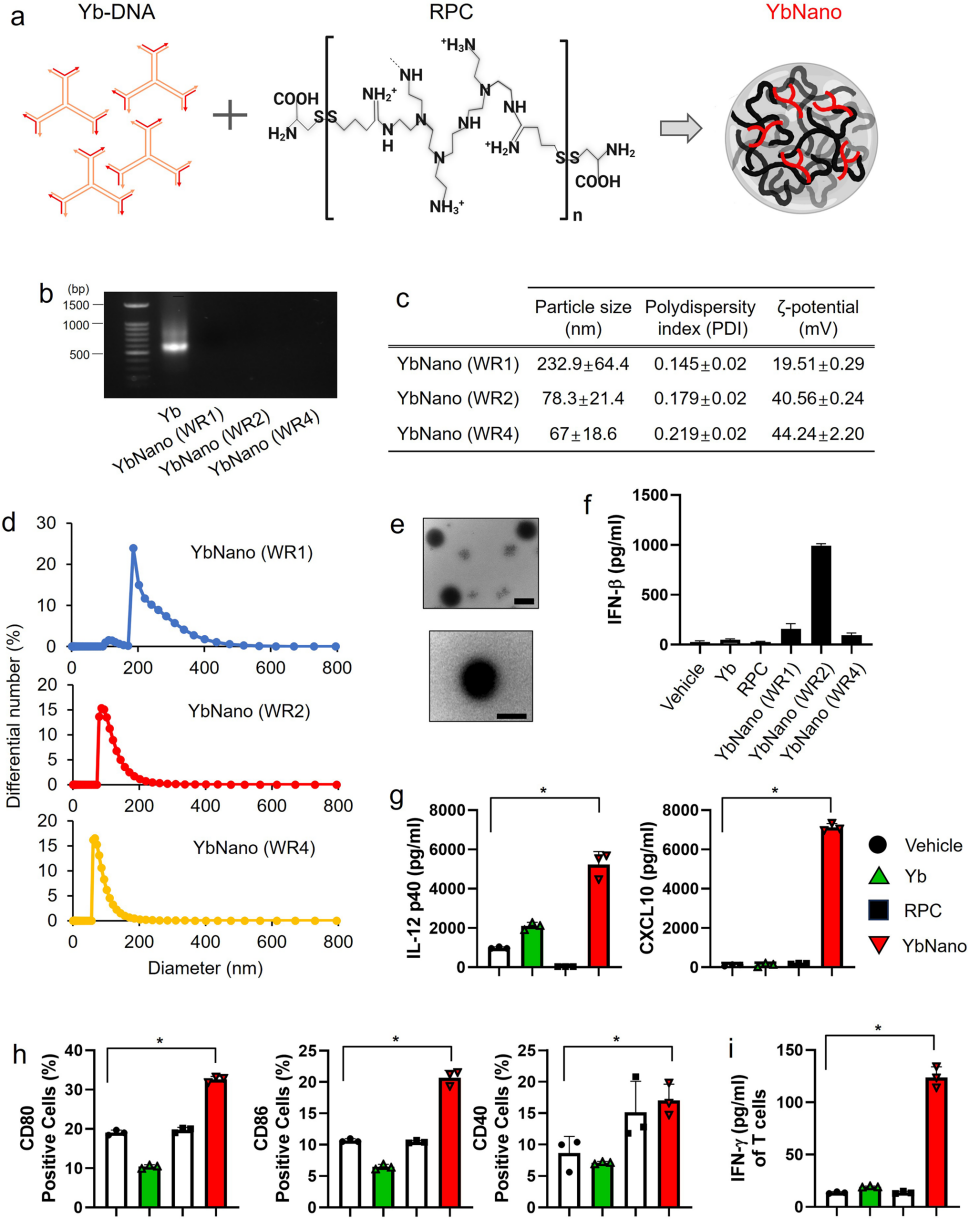
Characterization of Yb-DNA/RPC nanoparticles with immunostimulatory effects in dendritic cells. **a** Schematic illustration of Yb-DNA/RPC nanoparticles (YbNano) formation from branched Y-shaped DNA oligonucleotides (Yb) and reducible polycations-branched polyethyleneimine (RPC). **b** Agarose gel electrophoresis showing the complexation of Yb-DNA with RPC at three different weight ratios (WR; RPC weight per Yb-DNA weight). **c** Particle size, polydispersity index (PDI), and ζ-potential of YbNano (WR1, WR2, and WR4) were measured at pH 7.4. **d** Size distribution of three different YbNano at pH 7.4. **e** Transmission electron microscopy images of YbNano(WR2). Scale bar = 100 nm. **f** Bone marrow-derived dendritic cells (BMDCs) were treated with YbNano at different weight ratios for 18 h (Yb-DNA concentration fixed at 30 μg/ml), and protein levels of IFN-β in the culture supernatants were quantified by ELISA. **g **and **h** BMDCs were treated with vehicle, Yb-DNA (Yb, 8 μg/ml), RPC (16 μg/ml), or YbNano (WR2, Yb-DNA concentration of 8 μg/ml) for 18 h. **g** Protein levels of IL-12p40 and CXCL10 in the culture supernatants were measured by ELISA. **h** Protein expression of CD80, CD86, and CD40 was evaluated by flow cytometry. **i** BMDCs were stimulated with ovalbumin (10 μg/ml) in the presence of vehicle, Yb-DNA (Yb, 8 μg/ml), RPC (16 μg/ml), or YbNano (WR2, Yb-DNA concentration of 8 μg/ml for 18 h, followed by co-culture with CD8⁺ T cells for 3 days (DC:T cell ratio = 1:10). IFN-γ levels in the supernatant were measured by ELISA. Data are presented as mean ± SEM (n = 3 per group). *, *p* < 0.05

To develop immune-stimulatory oligonucleotides, the ability of several shapes of DNA oligonucleotides to induce the expression of immune cytokines was tested. These DNA oligonucleotides included Y-shaped DNA oligonucleotides (Ys), X-shaped DNA oligonucleotides (Xs), branched Y-shaped DNA oligonucleotides (Yb), and branched X-shaped DNA oligonucleotides (Xb) (Supplementary Fig. 2a and b). Among them, Yb-DNA showed the highest potential for immune-stimulatory activity (Supplementary Fig. 2c). To enhance cellular bioavailability, Yb-DNA was complexed with a nanoparticle carrier, RPC-bPEI_0.8kDa_ (reducible polycations-branched polyethyleneimine, RPC), to generate the Yb-DNA nanoparticle (YbNano) (Fig. 1a). Yb-DNA was complexed with RPC at three different weight ratios (WR), yielding WR1, WR2, and WR4, where the number indicates the reducible polycation weight per DNA weight. Gel electrophoresis results showed that YbNano formed complexes at various weight ratios (WRs) (Fig. 1b). Particle size, polydispersity index (PDI), and zeta-potential (ζ-potential) of YbNano with different WRs are shown in Fig. 1c and d. The size distribution of YbNano (WR2) is measured at mean diameter of 78.3 ± 21.4 nm and ζ-potential is 40.56 ± 0.24 mV (Fig. 1c and d). Transmission electron microscopy images revealed that YbNano formed uniform spherical nanoparticles with well-defined morphology (Fig. 1e).

Yb-DNA or RPC alone was not potent enough to induce a significant amount of IFN-β expression in mouse bone marrow-derived dendritic cells (BMDCs) while YbNano was able to considerably induce the expression of IFN-β (Fig. 1f), suggesting that complexing with RPC greatly enhanced the immune-stimulatory activity of Yb-DNA. Among the YbNano formulations with different WRs, WR2 showed the highest activity to induce IFN-β expression in BMDCs (Fig. 1f). Thus, YbNano with WR2 was chosen for further studies.

YbNano was able to induce the expression of other immune cytokines such as IL-12 compared to the vehicle, Yb-DNA, or RPC alone (Fig. 1g). Additionally, protein expression of leukocyte chemokines such as CXCL-10, was significantly increased in the YbNano group compared to Yb alone (Fig. 1g). A significant rise in the percentage of CD80 or CD86 positive cells and a mild increase in CD40 expression were observed in YbNano treated BMDCs measured by FACS analysis (Fig. 1h). Thus, YbNano exhibits significant immune-stimulatory activity in DCs, with increased expression of immune cytokines, leukocyte chemokines, and costimulatory molecules that can activate effector immune cells. Furthermore, DCs activated by YbNano were able to activate CD8^+^ T cells in a co-culture condition (Fig. 1i).

### Increased cellular bioavailability of YbNano as compared with Yb-DNA alone

**Fig. 2:**
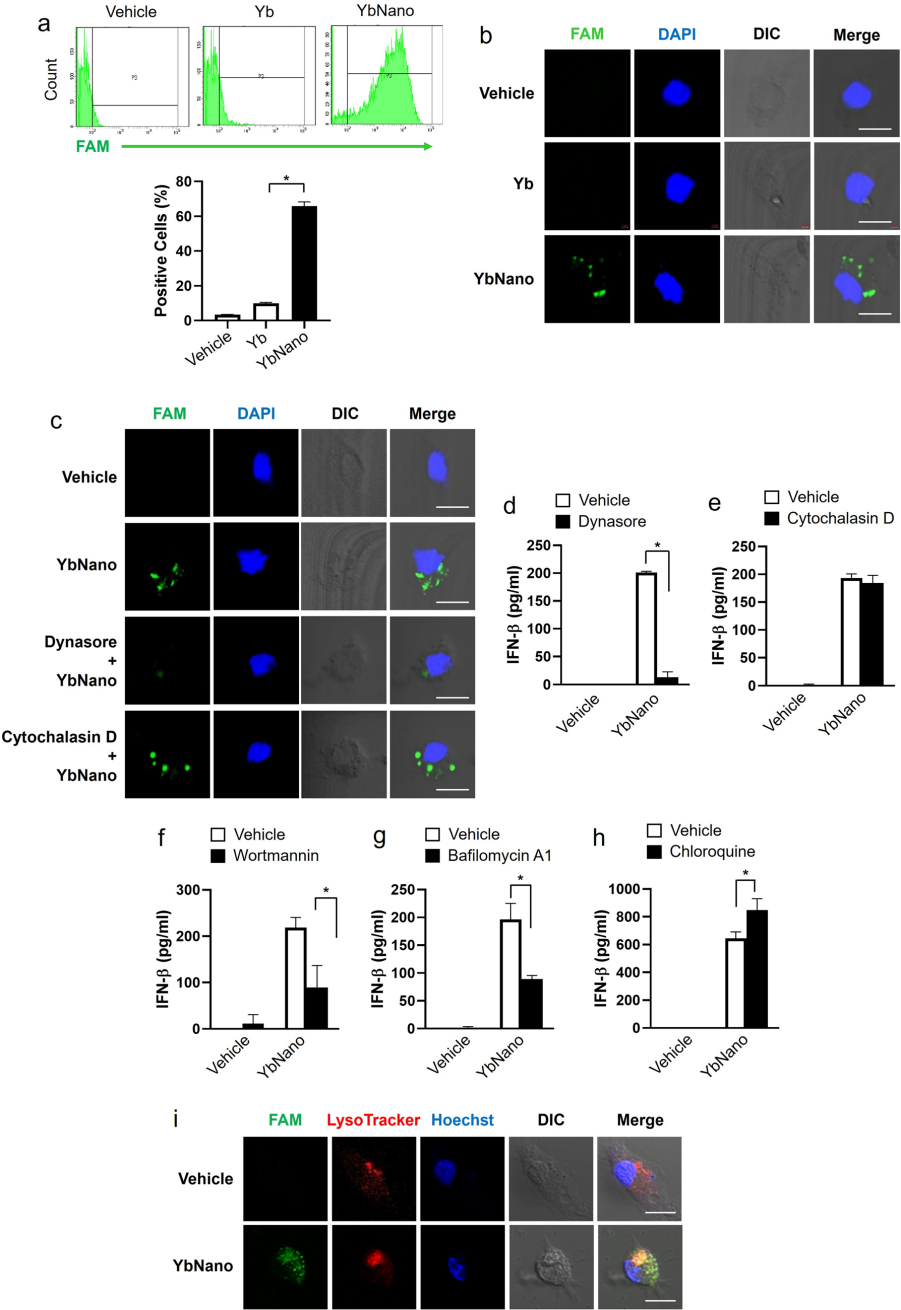
Intracellular uptake of Yb-DNA/RPC nanoparticles (YbNano) for immunostimulatory activity. **a** and **b** Bone marrow-derived dendritic cells (BMDCs) were treated with fluorescent Yb-DNA (8 μg/ml; green) or YbNano containing fluorescent Yb-DNA for 4 h. Intracellular uptake was analyzed using **a** flow cytometry and **b** confocal microscopy. **c**–**h** BMDCs were pretreated with dynasore (40 μM), cytochalasin D (40 μM), wortmannin (100 nM), bafilomycin A1 (50 nM), or chloroquine (20 μM) for 30 min, followed by treatment with YbNano (WR2, Yb-DNA concentration at 8 μg/ml) for 18 h. **c** Intracellular localization was evaluated by confocal microscopy, and **d**–**h** IFN-β production in the cell culture supernatants was assessed by ELISA. **i** BMDCs were treated with YbNano containing fluorescent Yb-DNA for 4 h. After the cells were further stained with LysoTracker (red) and Hoechst 33,342 (blue), the images were analyzed by confocal microscopy. Bar graph data are presented as mean ± SEM (n = 3 per group). *p < 0.05. Scale bar = 10 μm. *Yb* Yb-DNA; *DIC* differential interference contrast; FAM, carboxyfluorescein

To confirm that the cellular bioavailability was enhanced by RPC, we compared with cellular uptake of Yb-DNA and YbNano. Flow cytometry results showed that intracellular accumulation of Yb-DNA was dramatically increased when complexed with RPC as compared with Yb-DNA alone (Fig. 2a). Confocal analysis confirmed that complexation of Yb-DNA with RPC exhibited higher intracellular accumulation of Yb-DNA compared with Yb-DNA alone when using fluorescent Yb-DNA (Fig. 2b).

In order to identify the cellular uptake mechanism of YbNano, several inhibitors related to cell uptake were used to assess the immune-stimulatory activity of YbNano. DCs were treated with dynasore which is a clathrin- or caveolin-mediated endocytosis inhibitor and cytochalasin D which is an actin-dependent phagocytosis inhibitor, together with YbNano. Confocal analysis showed that dynasore greatly diminished cellular uptake of Yb-DNA while cytochalasin D did not inhibit cellular uptake of Yb-DNA (Fig. 2c). Consistently, IFN-β production increased by YbNano was significantly reduced by dynasore (Fig. 2d), while cytochalasin D did not suppress IFN-β production induced by YbNano (Fig. 2e). The results suggest that YbNano is likely to enter the DCs through clathrin- or caveolin-mediated endocytosis rather than phagocytosis.

To investigate the intracellular passage of YbNano after endocytosis, wortmannin, which inhibits the conversion of early endosomes to late endosomes, was used. Wortmannin reduced IFN-β production increased by YbNano (Fig. 2f), suggesting that the conversion to late endosomes may be a key pathway in the immune activity of YbNano. RPC has proton-buffering ability so that secondary or tertiary amines in RPC can escape the endosomes using osmotic pressure (Kang et al. 2011). YbNano has an amine group capable of accepting hydrogen ions. During endosome acidification, YbNano may accept the incoming hydrogen ions and form a buffer. Thus, the endosome is finally broken by the osmotic pressure of the continuously incoming hydrogen ions and other anions, yielding the escape of YbNano from the endosome. Treatment with bafilomycin A1 that inhibits endosome acidification decreased IFN-β production induced by YbNano (Fig. 2g), suggesting that endosome acidification is important for its escape. In contrast, chloroquine was used as an endosomal escape enhancer with its endosomolytic property since chloroquine can destabilize endolysosomal membranes to promote the release of co-delivered molecules to cytosol (Kim et al. 2018a; Hajimolaali et al. 2021; Choi et al. 2022; Cho et al. 2023; Debisschop et al. 2024; Liu et al. 2025). Chloroquine potentiated YbNano-induced IFN-β production (Fig. 2h), possibly because chloroquine facilitated YbNano escape from the endosome to cytosol. Confocal analysis showed that YbNano was partially co-localized in LysoTracker-positive compartments and partially distributed in the cytosol, suggesting its endosomal escape (Fig. 2i).

Thus, the results suggest that YbNano enters the DCs through a clathrin- or caveolin-mediated endocytosis pathway and that endocytosis process and endosomal acidification were important for immunostimulatory activity of YbNano.

### Enhanced therapeutic efficacy of checkpoint inhibition by YbNano in a mouse melanoma allograft model as a cancer immunotherapy

To investigate whether the immunostimulatory activity of YbNano could be used in cancer immunotherapy, a mouse melanoma allograft model was employed. First, an in vivo biodistribution study was performed to investigate the accumulation of Yb-DNA in tumors and other organs following administration in either nanoparticle formulation or non-nanoparticle formulation. Either YbNano containing Cy5.5-labeled Yb-DNA or Cy5.5-labeled Yb-DNA was intravenously administered to B16F10 allograft tumor-bearing mice (Supplementary Fig. 3a). At 48 h post-administration, fluorescence intensity in tumors from YbNano-treated mice was greater than that in tumors from Yb-DNA-treated mice (Supplementary Fig. 3b, c). Similarly, fluorescence intensities were higher in lung, heart, and liver of YbNano-treated mice than in those of Yb-DNA-treated mice, whereas in spleen and kidney, fluorescence intensities in Yb-DNA-treated mice were stronger than in YbNano-treated mice (Supplementary Fig. 3b, c). The results show that tumor accumulation of Yb-DNA was enhanced when it was administered in the nanoparticle formulation.

**Fig. 3:**
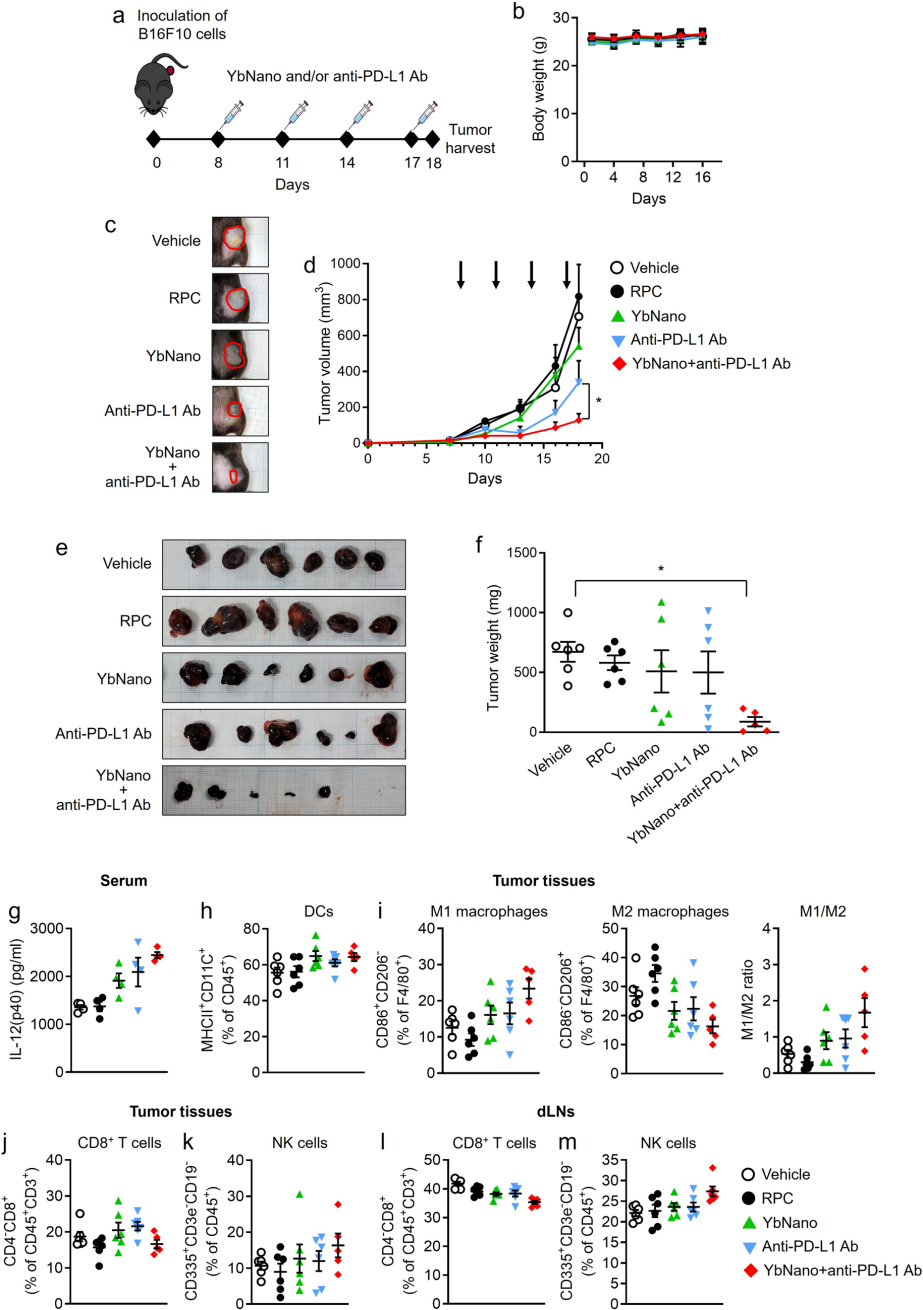
Enhanced anti-tumor efficacy of Yb-DNA/RPC nanoparticles (YbNano) in combination with an anti-PD-L1 antibody in a mouse allograft melanoma model. **a** Experimental scheme. C57BL/6 male mice were subcutaneously injected with B16F10 tumor cells. When tumors reached ~ 10 mm^3^, mice were treated four times at 3-day intervals with vehicle (30 μg IgG per mouse), RPC (20 μg per mouse), YbNano (WR2, Yb-DNA 10 μg per mouse), anti–PD-L1 antibody (Ab) (30 μg per mouse), or YbNano with anti–PD-L1 Ab. YbNano was intravenously injected and anti-PD-L1 antibody was intraperitoneally treated. Tumors were harvested 24 h after the final injection. **b** Body weights during the treatments. **c** Representative images of tumor-bearing sites in mice. **d** Tumor volumes were monitored over time. **e** Pictures of tumors isolated from each mouse. **f** Tumor weights were measured. **g** Serum IL-12 (p40) concentrations were measured by ELISA. **h**–**k** Flow cytometry analysis of immune cells in tumor tissues. **h** Dendritic cells (DCs). **i** M1 and M2 macrophages and the ratio of M1 to M2. **j** CD8⁺ T cells. **k** NK cells. **l** and **m** Flow cytometry analysis of CD8⁺ T cells and NK cells in draining lymph nodes (dLNs). **b **and **d** Data are presented as mean ± SEM (n = 5–6 mice per group). **f**–**m** Graphs are expressed with mean value and dots representing individual mouse (n = 5–6). *p < 0.05

Next, to investigate the efficacy of YbNano in cancer immunotherapy, when the tumor volume formed by subcutaneous inoculation of B16F10 melanoma cells on the left flank of the mice reached around 10 mm^3^, YbNano was intravenously injected 4 times, 3 days apart, with or without intraperitoneal administration of anti-PD-L1 antibody (Fig. 3a). There were no differences in the body weights between the groups during the treatments (Fig. 3b). The combination of YbNano with anti-PD-L1 antibody significantly increased the efficacy of the anti-PD-L1 antibody in reducing tumor volume compared with anti-PD-L1 antibody alone treatment (Fig. 3c, d). Combination of YbNano with anti-PD-L1 antibody exerted a significant reduction in the tumor size and weight compared with anti-PD-L1 antibody alone (Fig. 3e, f). To investigate in vivo immunostimulatory effects of YbNano, immune cell activation was analyzed in tumor tissues by flow cytometry. Serum IL-12 levels were elevated by YbNano treatment compared with vehicle or RPC alone (Fig. 3g). The mean value of YbNano with anti-PD-L1 Ab was higher than YbNano alone or anti-PD-L1 Ab alone (Fig. 3g). DC infiltration in tumor tissues was increased by YbNano or YbNano with anti-PD-L1 Ab compared with vehicle or RPC alone (Fig. 3h). YbNano treatment enhanced M1 macrophages, while it decreased M2 macrophages, compared with vehicle or RPC alone (Fig. 3i), showing the increased M1/M2 ratio. This tendency was more evident for YbNano with anti-PD-L1 Ab treatment, showing the increased M1 macrophages, the decreased M2 macrophages, and the increased M1/M2 ratio, compared with vehicle or RPC alone (Fig. 3i). CD8^+^ T cell infiltration was slightly higher in YbNano-treated tumors compared with RPC-treated tumors, whereas CD8^+^ T cell infiltration in YbNano with anti-PD-L1 Ab treatment group was similar with RPC treatment group (Fig. 3j). NK cell infiltration to tumor tissues in YbNano with anti-PD-L1 Ab group was the highest among the treatment groups (Fig. 3k). It was notable that NK cells were increased in draining lymph nodes by YbNano with anti-PD-L1 Ab treatment compared with other treatment groups, while CD8^+^ T cells in YbNano with anti-PD-L1 Ab treatment group were slightly decreased compared with other treatment groups (Fig. 3l, m). These results suggest that macrophages and NK cells may represent the primary effector populations contributing to antitumor immunity of YbNano in this model. Immunostaining of tumor tissues for F4/80 and NKp46 showed that the positive cells expressing F4/80 and NKp46 were significantly increased in the YbNano with anti-PD-L1 Ab group (Supplementary Fig. 4). A TUNEL assay to evaluate apoptosis showed enhanced tumor cell apoptosis in the YbNano with anti-PD-L1 Ab group (Supplementary Fig. 4).

These results suggest that treatment with YbNano increases the anti-cancer efficacy of the anti-PD-L1 antibody in a mouse melanoma allograft tumor model, supporting the potential usefulness of this combination therapy.

### Increased therapeutic efficacy by YbNano in the lung metastasis of breast tumors when used in combination therapy with doxorubicin or an anti-PD-L1 antibody

**Fig. 4:**
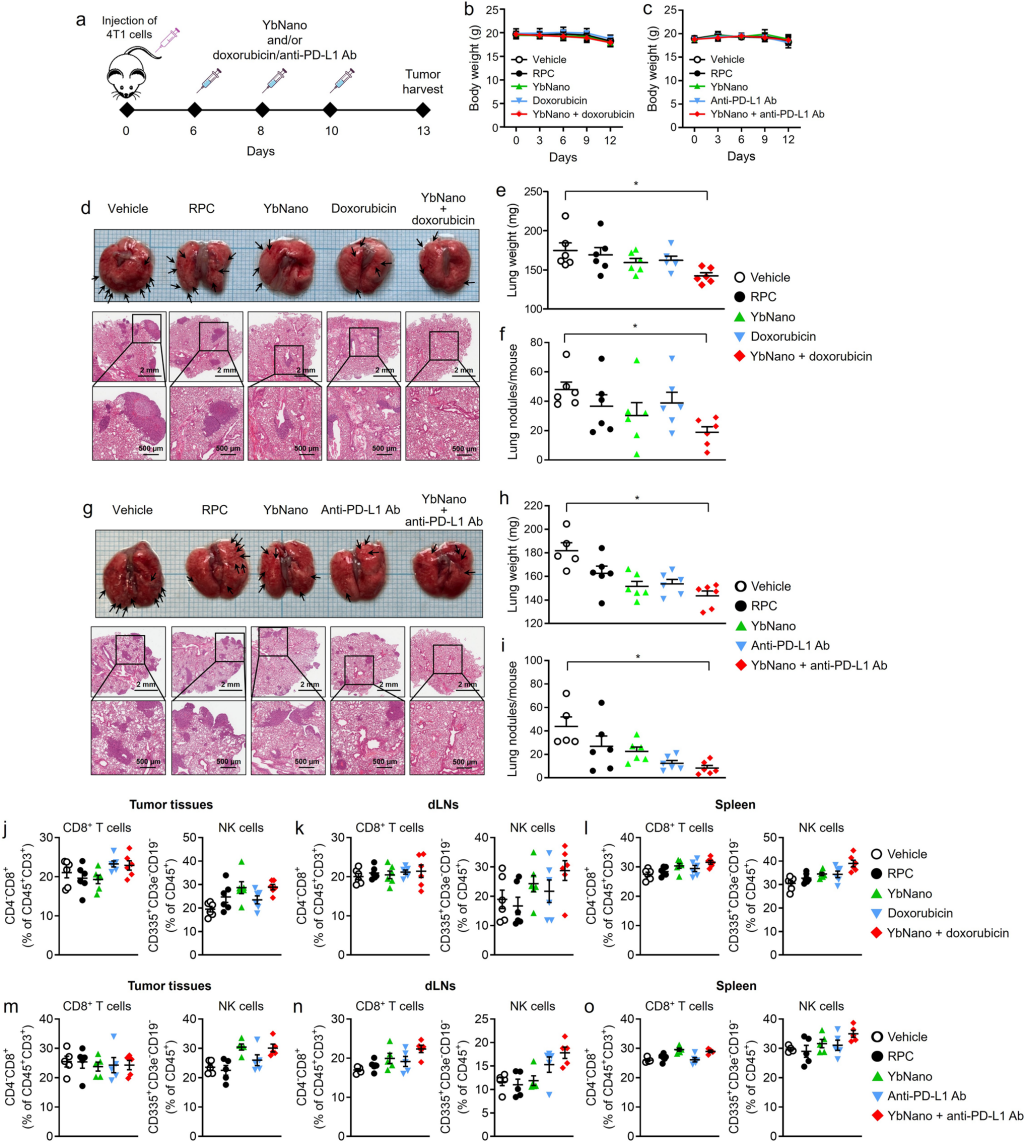
Therapeutic efficacy of Yb-DNA/RPC nanoparticles (YbNano) in combination with anti-PD-L1 antibody or chemotherapy in a mouse model of breast cancer lung metastasis. **a** Schematic illustration of the treatment schedule. Female BALB/c mice were intravenously injected with 4T1 breast cancer cells. On day 6 post-injection, mice were treated three times at 2-day intervals with vehicle (30 μg IgG per mouse), RPC (20 μg per mouse), YbNano (WR2, Yb-DNA 10 μg per mouse), doxorubicin (2 mg/kg), or YbNano with doxorubicin, anti–PD-L1 antibody (Ab) (30 μg per mouse), or YbNano with anti-PD-L1 Ab. YbNano was intravenously injected and doxorubicin or anti-PD-L1 Ab was intraperitoneally treated. Lungs were harvested 24 h after the final treatment. **b** Body weights of doxorubicin experiment groups. **c** Body weights of anti-PD-L1 Ab experiment groups. **d** Representative pictures of lung from each group in doxorubicin experiment and H&E staining picture of lung tissues. Metastatic nodules formed on the lung surface are shown (arrows). Scale bars represent 2 mm (upper) and 500 μm (lower). **e** Lung weights and **f** the number of metastatic nodules per mouse were quantified. **g** Representative pictures of lung from each group in anti-PD-L1 Ab experiment and H&E staining picture of lung tissues. Metastatic nodules formed on the lung surface are shown (arrows). Scale bars represent 2 mm (upper) and 500 μm (lower). **h** Lung weights and **i** the number of metastatic nodules per mouse were evaluated. **j**–**o** Flow cytometry analysis of CD8^+^ T cells and NK cells in tumor tissues, draining lymph nodes (dLNs), and spleen. **b** and **c** Data are presented as mean ± SEM (n = 5–6 mice per group). **e**, **f**, **h**, **i**–**o** Graphs are expressed with mean value and dots representing individual mouse (n = 4–5). *p < 0.05

To investigate whether YbNano would be beneficial for metastasis therapy, YbNano was applied to a mouse model of lung metastasis using aggressive 4T1 triple-negative breast cancer (TNBC) cells. YbNano was intravenously injected 3 times, 2 days apart, with or without doxorubicin or anti-PD-L1 antibody (Fig. 4a). There were no differences in the body weights between the groups during the treatments (Fig. 4b, c). Lung metastasis of 4T1 breast cancer cells was more effectively suppressed by the combination therapy of YbNano with doxorubicin (Fig. 4d). The combination treatment of YbNano and doxorubicin most potently and significantly reduced the lung weights compared with the vehicle group (Fig. 4e). The number of metastatic nodules in the lung was significantly lower in the YbNano with doxorubicin treatment group than that in the vehicle group (Fig. 4f). Similarly, lung metastasis of 4T1 breast cancer cells was more clearly reduced by the combination therapy of YbNano with anti-PD-L1 antibody (Fig. 4g). The combination treatment of YbNano with anti-PD-L1 antibody most effectively and significantly decreased the lung weights compared with the vehicle group (Fig. 4h). The number of metastatic nodules in the lung was the lowest in the YbNano with doxorubicin treatment group among all other groups (Fig. 4i). These results indicate that YbNano enhances anti-cancer effects against metastasis when used in combination with doxorubicin or an anti-PD-L1 antibody.

CD8^+^ T cells in tumor tissues were slightly increased in doxorubicin alone and YbNano with doxorubicin (Fig. 4j). The mean values of NK cells in tumor tissues were slightly higher in YbNano alone and YbNano with doxorubicin groups compared with vehicle or RPC group (Fig. 4j). CD8^+^ T cells in the draining lymph nodes were similar throughout the groups while the mean values of NK cells were elevated in YbNano alone and YbNano with doxorubicin groups compared with vehicle or RPC group (Fig. 4k). In the spleen, CD8^+^ T cell population showed a slight increasing trend in YbNano alone and YbNano with doxorubicin groups and the NK cell population was notably enhanced by YbNano with doxorubicin group (Fig. 4l). In Fig. 4m, CD8^+^ T cells in tumor tissues were similar throughout the groups while the mean values of NK cells in YbNano alone and YbNano with anti-PD-L1 Ab groups were elevated compared with vehicle or RPC group. The mean values of CD8^+^ T cells and NK cells in the draining lymph nodes were higher in the YbNano with anti-PD-L1 Ab group compared with other groups (Fig. 4n). In the spleen, CD8^+^ T cells were increased in YbNano alone and YbNano with anti-PD-L1 Ab groups compared with vehicle, RPC, or anti-PD-L1 Ab group (Fig. 4o). The mean value of NK cells in the YbNano with anti-PD-L1 Ab group was increased compared with other groups (Fig. 4o). In general, the increases in NK cell population by YbNano with doxorubicin or anti-PD-L1 Ab treatment were noted. These results suggest that increases in NK cells and, to a lesser extent, CD8⁺ T cells may contribute, at least in part, to enhanced tumor cell death, resulting in the suppression of tumor metastasis by combination therapy with YbNano.

### Dual activation of TLR9 and cGAS/STING pathway by YbNano for its immunostimulatory activity

**Fig. 5:**
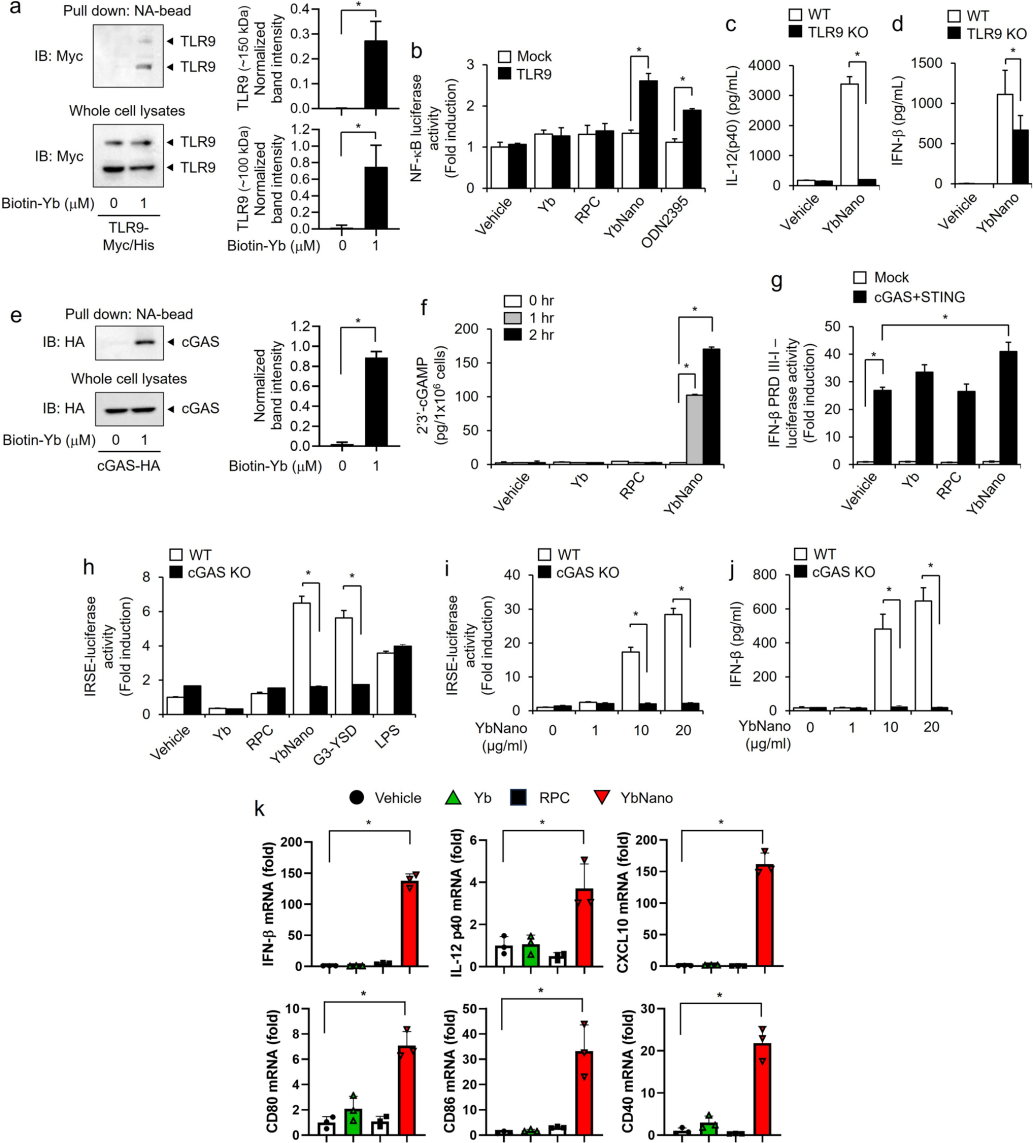
Dual activation of TLR9 and cGAS/STING by Yb-DNA. **a** After HEK293T cells were transfected with an expression plasmid of mTLR9-Myc/His, cell lysates were incubated with biotinylated Yb-DNA (biotin-Yb), followed by pulldown using NeutrAvidin(NA)-conjugated magnetic beads. Co-precipitation of TLR9 with Yb-DNA was analyzed by immunoblotting. TLR9 band intensities were evaluated using ImageJ software and pulldown protein band intensities were normalized by the corresponding protein band intensities in whole cell lysates. **b** After HEK293T cells were transfected with an expression plasmid of mTLR9 together with an NF-κB-driven luciferase reporter plasmid, cells were treated with vehicle, Yb-DNA (8 μg/ml), RPC (16 μg/ml), YbNano (WR2, Yb-DNA 8 μg/ml), or ODN2395 (1 µM) for 24 h. Luciferase activity was measured and expressed as a fold induction to vehicle group. **c**, **d** BMDCs derived from wild-type or TLR9-knockout (KO) mice were treated with YbNano (WR2, Yb-DNA 8 μg/ml). The expression of IL-12(p40) and IFN-β was quantified by ELISA. **e** After HEK293T cells were transfected with an expression plasmid of cGAS-3xHA, cell lysates were incubated with biotin-Yb, followed by pulldown using NA-conjugated magnetic beads. Co-precipitation of cGAS with Yb-DNA was evaluated by immunoblotting. cGAS band intensities were evaluated using ImageJ software and pulldown protein band intensities were normalized by the corresponding protein band intensities in whole cell lysates. **f** After BMDCs were treated with vehicle, Yb-DNA (8 μg/ml), RPC (16 μg/ml), or YbNano (WR2, Yb-DNA 8 μg/ml) for 0, 1, or 2 h, the production of 2′3′-cGAMP were quantified by ELISA. **g** After HEK293T cells were transfected with expression plasmids of cGAS and STING along with an IFN-β PRD III-I-luciferase reporter plasmid, cells were treated with vehicle, Yb-DNA (8 μg/ml), RPC (16 μg/ml), or YbNano (WR2, Yb-DNA 8 μg/ml) for 24 h. Luciferase activity was measured and expressed as a fold induction to vehicle group. **h** Wild-type (WT) THP-1 Lucia cells and cGAS knockout (KO) THP-1 cells were treated with vehicle, Yb-DNA (8 μg/ml), RPC (16 μg/ml), YbNano (WR2, Yb-DNA 8 μg/ml), G3-YSD (1 µg/ml), or LPS (10 ng/ml) for 24 h. Luciferase activity was measured and expressed as a fold induction to vehicle group. **i** and **j** THP-1 WT Lucia cells and cGAS KO THP-1 cells were treated with vehicle or YbNano (WR2, Yb-DNA concentration as indicated). **i** Luciferase activity was measured and expressed as a fold induction to vehicle group. **j** The expression of IFN-β was evaluated by ELISA. **k** BMDCs were treated with vehicle, Yb-DNA (Yb, 8 μg/ml), RPC (16 μg/ml), or YbNano (WR2, Yb-DNA 8 μg/ml) for 18 h. mRNA expression of indicated genes was analyzed by qRT-PCR. Bar graph data are presented as mean ± SEM (n = 3 per group). *p < 0.05. Yb, Yb-DNA

Next, to elucidate the cellular mechanism underlying the immune-stimulatory effects of YbNano, we investigated whether Yb-DNA acts as a ligand for immune receptors. Since DNA oligonucleotides are recognized by TLR9, leading to the activation of immune cells, we determined whether Yb-DNA could directly bind to TLR9 using an in vitro pull-down assay. TLR9 was co-precipitated with biotinylated Yb-DNA showing that Yb-DNA directly bound to TLR9 (Fig. 5a). To determine whether recognition of Yb-DNA by TLR9 is important for its immunostimulatory activity, we used both gain-of-function study and loss-of- function study approaches. YbNano increased NF-κB luciferase activity when TLR9 was overexpressed in HEK293T cells (Fig. 5b), while YbNano failed to increase the production of IL-12 in TLR9 knockout BMDCs (Fig. 5c and Supplementary Fig. 5a). The expression of IFN-β induced by YbNano was significantly reduced in TLR9 knockout BMDCs (Fig. 5d).

Since cGAMP synthase (cGAS) has been identified as a major cytoplasmic DNA sensor (Civril et al. 2013), we performed an in vitro pull-down assay to investigate whether Yb-DNA could directly bind to cGAS. cGAS was co-precipitated with biotinylated Yb-DNA showing the direct interaction between Yb-DNA and cGAS (Fig. 5e). cGAS activation by DNA culminates in 2′3′-cGAMP production. YbNano significantly increased the production of 2′3′-cGAMP in a time-dependent manner, indicating that YbNano induced the activation of cGAS (Fig. 5f). Overexpression of cGAS and STING significantly increased the IFN-β PRD III-I-luciferase relative activity in response to YbNano (Fig. 5g). In contrast, ISRE-dependent luciferase activity induced by YbNano was abolished in cGAS knockout THP-1 cells, just as ISRE-dependent luciferase activity increased by G3-YSD, a cGAS agonist, was diminished in cGAS knockout THP-1 cells (Fig. 5h and Supplementary Fig. 5b). ISRE-dependent luciferase activity increased by LPS, a TLR4 agonist, was not reduced in cGAS knockout cells (Fig. 5h). Furthermore, dose-dependent increases of ISRE-dependent luciferase activity and IFN-β expression by YbNano was abolished in cGAS knockout cells (Fig. 5i, j). In addition, cGAS knockdown by siRNA significantly reduced the IFN-β expression induced by YbNano compared to that of the negative control (Supplementary Fig. 5c).

Receptor-mediated expression of immune mediators is dependent on transcriptional regulation. Thus, we investigated whether YbNano regulated the expression of immune cytokines and mediators at the mRNA levels. YbNano increased the mRNA levels of immune cytokines such as IFN-β and IL-12, and a chemokine such as CXCL10, and co-stimulatory molecules such as CD80, CD86, and CD40 in BMDCs (Fig. 5k). The results suggest that the activation of the immune receptor by YbNano culminates in the activation of cellular signaling pathways, leading to transcriptional activation. Collectively, these results indicate that YbNano exerted its immune stimulatory activity through the dual activation of TLR9 and cGAS/STING.

## Discussion

The results of our study present YbNano as an integrated immunostimulatory agent, possibly beneficial for combination cancer therapy, mediated through dual engagement of TLR9 and cGAS/STING pathway. Approaches that activate innate immune receptors such as cGAS/STING or TLR9 for antitumor immunotherapy have been widely investigated (Weiner et al. 1997; Seok et al. 2023). Most of these strategies have focused on targeting a single immune receptor. Even in studies aiming to achieve dual immune activation, multiple receptors are typically targeted through the combined use of individual agonists specific to each receptor. For example, Hajiabadi et al. simultaneously administered a STING agonist and a TLR9 agonist to achieve synergistic therapeutic efficacy in a colon carcinoma model (Hajiabadi et al. 2023). In contrast, Yb-DNA is capable of activating both endolysosomal TLR9 and cytosolic cGAS/STING, possibly promoting synergistic activation of immune responses. TLR9 is localized in the endoplasmic reticulum of dendritic cells and macrophages and, in response to ligand stimulation, translocates to endolysosomal or lysosomal compartments, where it binds to the ligand (Latz et al. 2004; Heinz et al. 2020)**.** Our confocal analysis shows that Yb-DNA was partially co-localized with LysoTracker, a lysosomal compartment marker, and partially observed in cytosol, suggesting that Yb-DNA can be localized in both lysosome and cytosol. These results further suggest that the RPC-bPEI nanocomplex delivers Yb-DNA, at least in part, to lysosomal compartments, as well as the cytosol. In addition, results from knockout studies support the involvement of two receptors in immunostimulatory property of YbNano. IFN-β production induced by YbNano was not completely abolished in TLR9 knockout cells, suggesting the possibility of an additional pathway. IFN-β production induced by YbNano was markedly reduced in cGAS knockout cells, suggesting the role of cGAS in Yb-DNA-induced IFN-β production. Collectively, our results suggest that Yb-DNA engages both TLR9 and cGAS in different locations, when introduced in the form of nanocomplex with RPC-bPEI. Yet, the relative contribution of each pathway to the immunostimulatory activity of Yb-DNA remains to be fully resolved.

The combination of Yb-DNA with RPC-bPEI_0.8kDa_ represents a rationally engineered and optimized platform for developing nucleic acid-based immunostimulatory agents. Conventional low molecular weight bPEI_0.8kDa_ is non-toxic but insufficient for stable DNA condensation, whereas higher molecular weight PEI increases cytotoxicity (Kang et al. 2011). Incorporation of disulfide bonds into bPEI_0.8kDa_ generated RPC-bPEI_0.8kDa_, which forms stable polycation/pDNA complexes while maintaining low cytotoxicity (Kang et al. 2011). Its reducible architecture allows intracellular degradation by glutathione, restoring the non-toxic polymer post-delivery. This design ensures extracellular stability, promotes endosomal escape, and maximizes exposure of DNA cargos to both TLR9 and cGAS, thereby synergistically enhancing immune activation. The exclusion of YbNano (WR1) due to heterogeneous particle size further underscores the importance of optimizing nanoparticle uniformity for reproducible biological outcomes. The RPC-bPEIs were designed to release the DNA under thiol/polyanion-rich environments like cytosol (Kang et al. 2011). To determine Yb-DNA release from the nanocomplex, the thiol-rich environment (about 20 mM) of the cytosol was mimicked with dithiothreitol (DTT, a reducing reagent) and a model polyanion (heparin) was added to mimic intracellular environments (higher polyanion concentrations) as described in a previous report (Kang et al. 2011). After incubation of YbNano with heparin and DTT, the migration of the released Yb-DNA from nanocomplex was compared with native Yb-DNA at an equivalent amount (Supplementary Fig. 6). The results showed that (1) incubation of YbNano with heparin and DTT resulted in the release of Yb-DNA, and (2) the released Yb-DNA migrated to the same position as native Yb-DNA, suggesting that Yb-DNA released from the nanocomplex maintained the shape. Approximately 82% of Yb-DNA was released from the RPC-bPEI_0.8kDa_ complex upon treatment with heparin and DTT, suggesting that substantial amount of Yb-DNA could be released from the nanoparticle complex under thiol- and polyanion-rich environments such as cytosol.

The DNA sequence, especially the presence of CpG motifs, can be the fundamental determinant of TLR9 activation. We compared Yb-DNA with and without CpGs in order to determine the importance of CpG motifs in the sequence. Yb-DNA with CpGs (WR2) showed about 3-times higher activity to induce IFN-β production than Yb-DNA without CpGs (WR2) in mouse primary dendritic cells (Supplementary Fig. 7). The results show that the presence of CpG motifs is important to confer immunostimulatory activity. Yamamoto et al. (2000) identified key characteristics of immunostimulatory DNA sequences in their research on Mycobacterium bovis BCG (Yamamoto et al. 2000). The immunostimulatory properties of DNA sequences were dependent on the presence of specific palindromic sequences and CpG motifs. Oligodeoxynucleotides (ODNs) containing palindromic sequences such as 5′-GACGTC-3′, 5′-AGCGCT-3′, and 5′-AACGTT-3′ significantly promoted NK cell activity and IFN induction. In contrast, sequences of the same length without palindromic structures exhibited minimal immunostimulatory activity (Yamamoto et al. 2000). Small alterations in the sequence, such as exchanging two bases within an active palindromic structure (for example, swapping G and T within GACGTC), led to a substantial reduction in immune activity. Additionally, oligo-guanylate (oligo-G) sequences also interacted with the core palindromic sequence to further enhance the immunostimulatory activity (Yamamoto et al. 2000). CpG ODNs are categorized into three classes (Class A, B, and C) based on their sequence characteristics and backbone modifications (Krieg 2006). Class A CpGs require the formation of higher-order structures (e.g., G-quadruplexes or multimeric aggregates) to localize to early endosomes and induce robust IFN-α production, whereas linear Class B CpGs typically localize to late endosomes (Verthelyi et al. 2001; Krieg 2006). In addition to sequence, several studies suggest that assembling ODNs into defined nanostructures confers enhanced immunostimulatory activity, particularly TLR9 activation, compared with linear counterparts (Roberts et al. 2005). Nishikawa et al. (2008) showed that immunostimulatory activity was enhanced by complexing CpG ODN into a Y-shaped structure compared with single- or double-stranded ODN (Nishikawa et al. 2008). Consistently, our results in previous study, showed that ligated form of Y-shaped or X-shaped DNA exhibited immunostimulatory efficacy in dendritic cells, inducing the expression of cytokines and co-stimulatory molecules, mediated by direct association with TLR9 (Koo et al. 2015; Yang et al. 2019). In addition, various ODN nanostructures containing CpG motifs, such as RNA triangle nanoparticles (Khisamutdinov et al. 2014), DNA tetrahedra (Li et al. 2011), DNA origami structures (Schuller et al. 2011), DNA polypod-like structures (Mohri et al. 2012), DNA tetrahedrons (Sadowski et al. 2014), and DNA dendrimers (Mohri et al. 2015) were reported to exhibit immunostimulatory activity. These studies support the notion that structured ODN assemblies, including our Yb-DNA, confer immunostimulatory properties. Our results suggest that both structural architecture and CpG sequence contribute to the immunostimulatory activity of Yb-DNA. While Supplementary Fig. 2 indicates that the branched structure can influence the immunostimulatory activity of DNA oligonucleotides, Supplementary Fig. 7 shows the importance of CpG motifs. However, the relative contribution of these two factors has not yet been quantitatively dissected.

Induction of CD8^+^ T cell and NK cell responses can limit tumor progression with cytotoxic killing of tumor cells. Intratumoral injection of a CpG ODN promoted infiltration and expansion of CD8^+^ T cells, thereby reversing resistance to PD-1 blockade in mice transplanted with the CT26 colon carcinoma cells (Wang et al. 2016). In addition, TLR9 activation by CpG ODNs in dendritic cells resulted in the production of IFNs, subsequently leading to activation of NK cells that were capable of direct tumor killing during the CpG ODN-induced antitumor response (Chuang et al. 2020). cGAS/STING pathway is considered effective to induce the activation of NK cells for an anti-tumor activity by producing IFNs. Tumor cell-secreted cGAMP induced the activation of NK cell response, mediated through STING-dependent IFNs response in non-tumor cells in tumor microenvironment (Marcus et al. 2018). In contrast, the influences of cGAS/STING in the activation of CD8^+^ T cells are rather complex. cGAS/STING activation compromised the function and proliferation of CD8^+^ T cells, leading to increased cell death (Kuhl et al. 2023). Robust activation of STING resulted in cell death of CD4^+^ and CD8^+^ T cells (Gulen et al. 2017). Sivick et al. reported that the magnitude of STING activation exhibited distinct effector responses that lower dose of STING agonist led to optimal CD8^+^ T cell responses while higher dosing compromised effective anti-tumor immunity, suggesting optimal immunogenic dosage was required for combination therapy (Sivick et al. 2018). Our results from a melanoma allograft mouse model and two metastasis mouse models suggest that NK cells and, to a lesser extent, CD8⁺ T cells may contribute, at least in part, to the anti-tumor effects of Yb-DNA. Since Type I IFNs are known inducers of NK cell activation, IFN-β produced by Yb-DNA in dendritic cells may act as an effector to promote the activation of NK cells.

In addition to immunotherapy, a variety of strategies for cancer treatment are currently being pursued. The approaches include drug repurposing of GLP-1 receptor agonists (Valencia-Rincon et al. 2025), targeting the 20S proteasome (Atta et al. 2023), as well as the use of natural compounds such as hinokitiol (Chiang et al. 2024). Valencia-Rincón et al. reported that GLP-1 receptor agonists may lower cancer incidence risk in some clinical cases and that several preclinical cancer studies showed anticancer effects of GLP-1 receptor therapies, potentially mediated through direct action on immune cells or modulation of metabolic status (Valencia-Rincon et al. 2025). In addition, targeting the 20S proteasome complex using synthetic drugs and natural products has been proposed as an anticancer strategy (Atta et al. 2023). Furthermore, inhibition of stemness-progression of cancer cells by natural product such as hinokitiol has been shown to be beneficial for preventing tumor relapse or metastasis (Chiang et al. 2024). Integrating nucleic acid-based immune-stimulatory platform presented in our study with other anticancer approaches may provide a promising strategy to achieve more sustainable cancer treatments that effectively promote tumor regression.

RPC-bPEI_0.8kDa_, which was used as a carrier system, exhibited lower cytotoxicity than higher molecular weight PEI. In addition, the use of RPC-bPEI_0.8kDa_ promoted tumor accumulation of Yb-DNA. Nevertheless, in vivo administration of polycationic polymer may still raise potential concerns regarding systemic toxicity. Therefore, comprehensive safety evaluation will be necessary in future studies to further support the translational potential of this platform. Furthermore, while our study demonstrated that YbNano effectively suppressed tumor growth in mouse allograft and metastasis models, the current study primarily evaluates short-term tumor growth inhibition. Additional experiments such as survival analysis, tumor rechallenge studies, or long-term immune memory evaluation would further strengthen the therapeutic potential of YbNano.

Collectively, YbNano enhances anti-cancer immunity via dual activation of TLR9 and cGAS/STING, synergizing with checkpoint blockade and chemotherapy. Our study suggests that YbNano represents a rationally engineered and optimized nucleic acid-based immunotherapeutic agent that may be beneficial for combination cancer therapy by modulating the tumor microenvironment to be favorable for anticancer therapy.

## Supplementary Information

Below is the link to the electronic supplementary material.

Supplementary file (PDF 907 KB)

## Data Availability

The data that support the findings of this study are available from the corresponding author upon reasonable request.
