# Supplementary material for: Nanostructured branched Y-DNA promotes antitumor immunity through dual activation of cGAS/STING and TLR9
Source: Arch Pharm Res. 2026 Mar 18;49(3):321–38. doi: 10.1007/s12272-026-01607-y (PMC13076375; doi:10.1007/s12272-026-01607-y)
Supplement: Supplementary file 1 — Supplementary file (PDF 907 KB) [file 12272_2026_1607_MOESM1_ESM.pdf]

Supplementary Materials

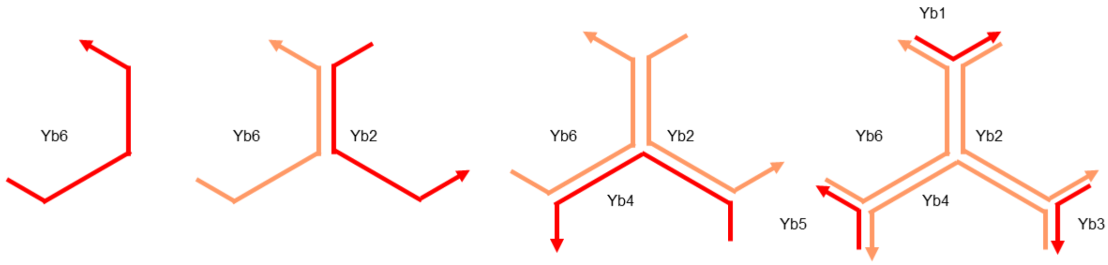

Yb1	GTCGCTGACACTGTGATGGGATTGTAGGTGAACGCT
Yb2	AGCGTTCACCTACAATCCAGGAGTCACGCTTACAGAATCTATGTCCGGTAGTGAAATGAGAAC GATCTCTGAGGTGGATCTCGGACATGAGTTCAGAGCGTAAAGGTT
Yb3	AACCTTTACGCTCTGAACCATACCTGTCGACTTGAA
Yb4	TTCAAGTCGACAGGTATGTCATGTCCGAGATCCACCTCAGAGATCGTTCTCATTGGAGTAACG GTCTGTGTCTGACCTATAGCTTGTCTGGTCCAGTGCTGTGTCCTT
Yb5	AAGGACACAGCACTGGACATTTGTTCGTGGAGACTT
Yb6	AAGTCTCCACGAACAAATCAGACAAGCTATAGGTCAGACACAGACCGTTACTCCTCACTACC GGACATAGATTCTGTAAGCGTGACTCCTCATCACAGTGTCAGCGAC

Supplementary Figure 1. The synthesis steps and oligodeoxynucleotide sequences of the branched Y-shape DNA (Yb-DNA). Yb-DNA was formed by mixing equal molar amounts of Yb1, Yb2, Yb3, Yb4, Yb5, and Yb6 oligonucleotide strands.

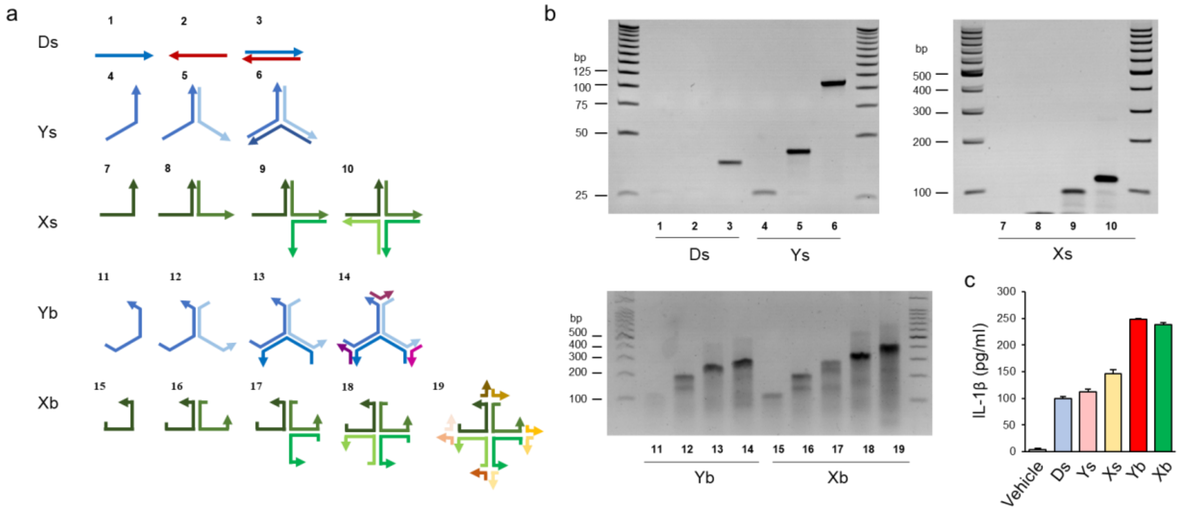

d

**Ds**	
Ds1	ATCTATGTCTGGTAGTGAAATGAGACAGATCTCTGA
Ds2	TCAGAGATCTGTCTCATTTCACTACCAGACATAGAT
**Ys**	
Ys1	ATCTATGTCCGGTAGTGAAATGAGAACGATCTCTGA
Ys2	TCAGAGATCGTTCTCATTGGAGTAACGGTCTGTGTC
Ys3	GACACAGACCGTTACTCCTCACTACCGGACATAGAT
**Xs**	
Xs1	ATCTATGTCCGGTAGTGAAATGAGAACGATCTCTGA
Xs2	TCAGAGATCGTTCTCATTGGAGTAACGGTCTGTGTC
Xs3	GACACAGACCGTTACTCCCACTCATCGCTGACAACT
Xs4	AGTTGTCAGCGATGAGTGTCACTACCGGACATAGAT
**Xb**	
Xb1	GTCGCTGACACTGTGATGGGATTGTAGGTGAACGCT
Xb2	AGCGTTCACCTACAATCCAGGAGTCACGCTTACAGAATCTATGTCCGGTAGTGAAATGAGAA CGATCTCTGAGGTGGATCTCGGACATGAGTTCAGAGCGTAAAGGTT
Xb3	AACCTTTACGCTCTGAACCATACCTGTCGACTTGAA
Xb4	TTCAAGTCGACAGGTATGAATACACAGCATGTTCTT
Xb5	AAGAACATGCTGTGTATTTCATGTCCGAGATCCACCTCAGAGATCGTTCTCATTGGAGTAACG GTCTGTGTCTGACCTATAGCTTGTCTGGTCCAGTGCTGTGTCCTT
Xb6	AAGGACACAGCACTGGACTGGACCATTGCTTGAGTA
Xb7	TACTCAAGCAATGGTCCAATTTGTTCGTGGAGACTT
Xb8	AAGTCTCCACGAACAAATCAGACAAGCTATAGGTCAGACACAGACCGTTACTCCCACTCATC GCTGACAACTCATCAGTTGCTTCTATGAGTCAGTACGCAGAGTGAA
Xb9	TTCACTCTGCGTACTGACCATTATCCGATATCACTT
Xb10	AAGTGATATCGGATAATGTCAGAATAGCAATTAGTT
Xb11	AACTAATTGCTATTCTGATCATAGAAGCAACTGATGAGTTGTCAGCGATGAGTGTCACTACCG GACATAGATTCTGTAAGCGTGACTCCTAATTGAGTCGGATACCTT
Xb12	AAGGTATCCGACTCAATTCATCACAGTGTCAGCGAC

Supplementary Figure 2. Structures and immunological activity of various DNA oligonucleotide types. (a) Schematic diagrams of various DNA types; double-stranded DNA (Ds), Y-shaped DNA (Ys), X-shaped DNA (Xs), branched Y-shape DNA (Yb), and branched X-shape DNA (Xb). (b) Each oligonucleotide synthesized at various stages was analyzed

using agarose gel electrophoresis. (c) Mouse bone marrow-derived dendritic cells were treated with Ds, Ys, Xs, Yb, or Xb for 24 h. IL-1β levels in the supernatant were measured by ELISA. Bar graph data are presented as mean ± SEM (n = 3 per group). (d) Sequences of Ds, Ys, Xs, and Xb.

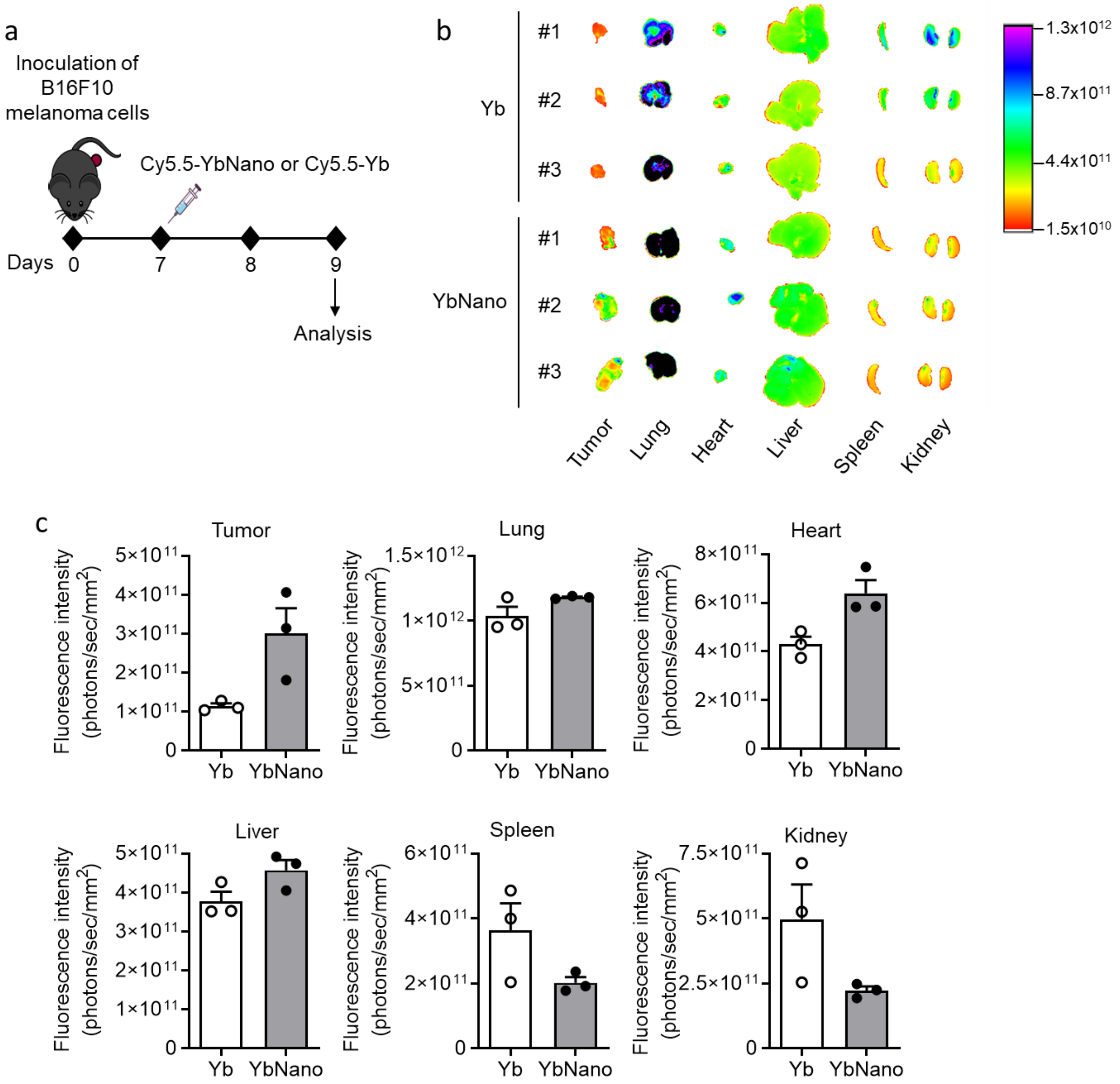

Supplementary Figure 3. ***In vivo*** **biodistribution of Yb-DNA in nanoparticle formulation and non-nanoparticle formulation.** (a) Experimental scheme. B16F10 melanoma cells were subcutaneously injected into the flanks of C57BL/6 male mice. After 7 days, the mice were intravenously administered with either Cy5.5-labeled Yb-DNA or YbNano containing Cy5.5-labeled Yb-DNA (40 µg Yb-DNA per mouse) via the tail vein. At 48 h post-administration, tumor and other major organs such as lung, heart, liver, spleen, and kidney were harvested. Fluorescence images of the organs were acquired using an *in vivo* Xtreme imaging system

with excitation/emission at 670/700 nm. The fluorescence intensity was quantified as the mean photon flux (photons/sec/mm^2). (b) Representative fluorescence images of tumor, lung, heart, liver, spleen, and kidney. The # means each mouse. (c) The fluorescence intensities in the organs are presented as mean ± SEM (n=3 mice per group).

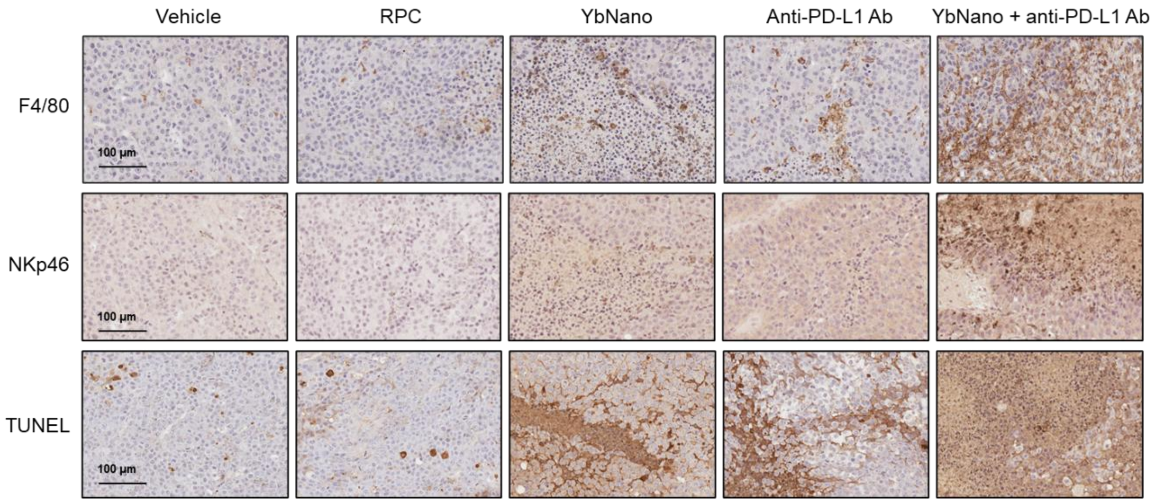

Supplementary Figure 4. Histological analysis of tumor tissues obtained from a mouse allograft melanoma model. Tumor tissues were obtained from mice described in Figure 3. Tumor sections were immunostained for F4/80 and NKp46, and subjected to TUNEL assay. Scale bars represent 100 µm.

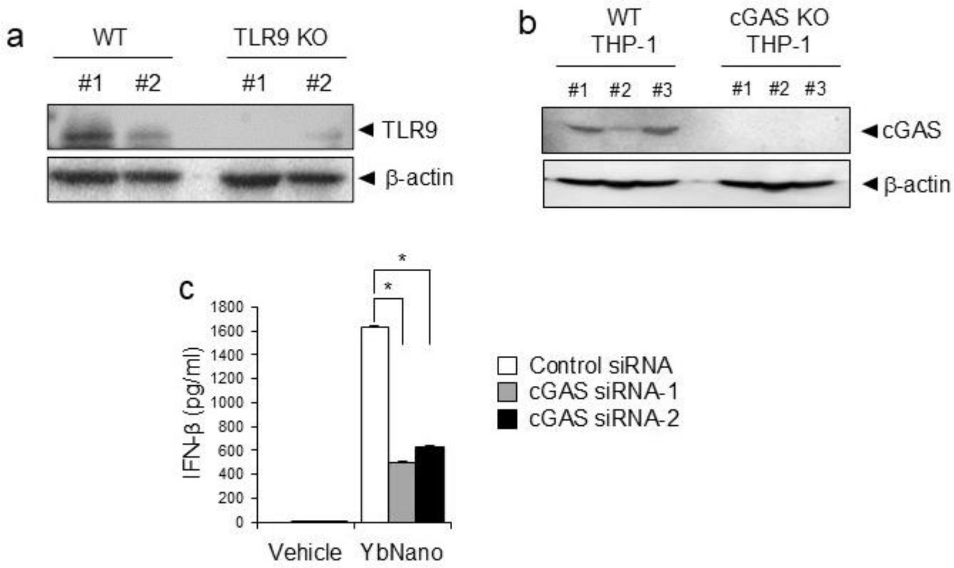

Supplementary Figure 5. Verification of TLR9 and cGAS expression. (a) TLR9 expression was examined in bone marrow-derived dendritic cells from wild-type or TLR9 knockout (KO) mice. Protein levels of TLR9 were analyzed by immunoblotting. (b) cGAS expression was investigated using THP-1 Lucia ISG (WT) and THP-1 cGAS knockout (KO) cells. Protein levels of cGAS were analyzed by immunoblotting. (c) Bone marrow-derived dendritic cells were transfected with siRNA targeting cGAS, followed by treatment with vehicle or YbNano (WR2, Yb-DNA 8 μg/ml) for 18 h. IFN-β production in the cell culture supernatants was assessed by ELISA. Data are presented as mean ± SEM (n = 3 per group). *, $p < 0.05$.

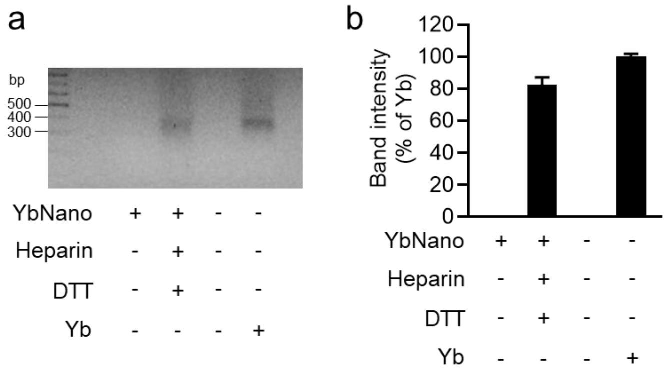

Supplementary Figure 6. Yb-DNA release from the nanocomplex. After YbNano (WR2, Yb-DNA concentration of 16 μg/ml) was incubated with heparin (100 μg/ml) and DTT (20 mM) for 24 h, the reaction mixtures were resolved on agarose gel to analyze the migration of Yb-DNA. (a) Representative agarose gel image. Lane 1: YbNano alone; Lane 2: YbNano treated with heparin and DTT; Lane 3: vehicle; Lane 4: Yb-DNA (Yb, 16 μg/ml). (b) The band intensities of Yb-DNA were measured and expressed as a percentage of Yb-DNA (Lane 4). Data are presented as mean ± SEM (n = 3).

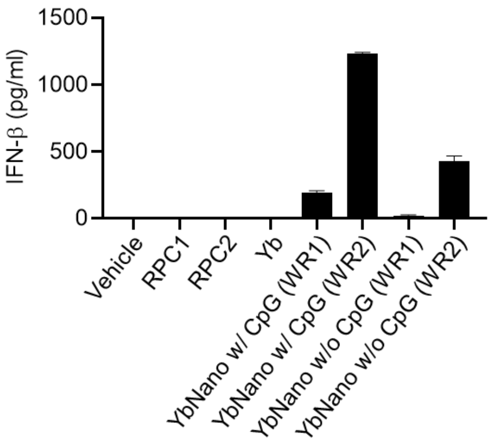

Supplementary Figure 7. Comparison of immunostimulatory activity between Yb-DNA with and without CpG motifs. Bone marrow-derived dendritic cells were treated with vehicle, RPC1 (20 μg/ml), RPC2 (40 μg/ml), Yb-DNA (Yb, 20 μg/ml), and YbNano with or without CpGs (WR1 or WR2; 20 μg/ml of Yb-DNA) for 18 h. The production of IFN-β was evaluated by ELISA. Data are presented as mean ± SEM (n = 3).
